# Genetic Polymorphisms Affecting IDO1 or IDO2 Activity Differently Associate With Aspergillosis in Humans

**DOI:** 10.3389/fimmu.2019.00890

**Published:** 2019-05-07

**Authors:** Valerio Napolioni, Marilena Pariano, Monica Borghi, Vasilis Oikonomou, Claudia Galosi, Antonella De Luca, Claudia Stincardini, Carmine Vacca, Giorgia Renga, Vincenzina Lucidi, Carla Colombo, Ersilia Fiscarelli, Cornelia Lass-Flörl, Alessandra Carotti, Lucia D'Amico, Fabio Majo, Maria Chiara Russo, Helmut Ellemunter, Angelica Spolzino, Paolo Mosci, Stefano Brancorsini, Franco Aversa, Andrea Velardi, Luigina Romani, Claudio Costantini

**Affiliations:** ^1^Department of Neurology and Neurological Sciences, School of Medicine, Stanford University Stanford, CA, United States; ^2^Department of Experimental Medicine, University of Perugia, Perugia, Italy; ^3^Unit of Endocrinology and Diabetes, Bambino Gesù Children's Hospital, Rome, Italy; ^4^Fondazione IRCCS Ca' Granda, Ospedale Maggiore Policlinico, University of Milan, Milan, Italy; ^5^Bambino Gesù Children's Hospital IRCCS, Rome, Italy; ^6^Division of Hygiene and Medical Microbiology, Innsbruck Medical University, Innsbruck, Austria; ^7^Institute of Hematology-Centro di Ricerche Emato-Oncologiche, University of Perugia, Perugia, Italy; ^8^CF Centre, Medical University Innsbruck, Innsbruck, Austria; ^9^Division of Hematology, Azienda Ospedaliero-Universitaria di Parma, Parma, Italy; ^10^Department of Veterinary Medicine, University of Perugia, Perugia, Italy

**Keywords:** IDO1, IDO2, aspergillosis, cystic fibrosis, hematopoietic stem cell transplantation

## Abstract

*Aspergillus* is the causative agent of human diseases ranging from asthma to invasive infection. Genetic and environmental factors are crucial in regulating the interaction between the host and *Aspergillus*. The role played by the enzyme indoleamine 2,3-dioxygenase 1 (IDO1), which catalyzes the first and rate-limiting step of tryptophan catabolism along the kynurenine pathway, is increasingly being recognized, but whether and how genetic variation of IDO1 influences the risk of aspergillosis in susceptible patients is incompletely understood. In addition, whether the closely related protein IDO2 plays a similar role remains unexplored. In the present study, we performed genetic association studies in two different cohorts of susceptible patients [cystic fibrosis (CF) patients and recipients of hematopoietic stem cell transplantation (HSCT)], and identified *IDO1* polymorphisms that associate with the risk of infection in both cohorts. By using human bronchial epithelial cells and PBMC from CF and HSCT patients, respectively, we could show that the *IDO1* polymorphisms appeared to down-modulate IDO1 expression and function in response to IFNγ or *Aspergillus* conidia, and to associate with an increased inflammatory response. In contrast, *IDO2* polymorphisms, including variants known to profoundly affect protein expression and function, were differently associated with the risk of aspergillosis in the two cohorts of patients as no association was found in CF patients as opposed to recipients of HSCT. By resorting to a murine model of bone marrow transplantation, we could show that the absence of IDO2 more severely affected fungal burden and lung pathology upon infection with *Aspergillus* as compared to IDO1, and this effect appeared to be linked to a deficit in the antifungal effector phagocytic activity. Thus, our study confirms and extends the role of IDO1 in the response to *Aspergillus*, and shed light on the possible involvement of IDO2 in specific clinical settings.

## Introduction

*Aspergillus* species are environmental fungi to which humans are constantly exposed and the inability to properly control the invasion results in a wide spectrum of diseases, ranging from asthma to the more severe and life-threatening invasive aspergillosis (IA) (1). The host immune response as well as the degree of exposure are critical determinants for the development of infection and gauge the clinical manifestations of aspergillosis. Indeed, specific diseases and/or clinical conditions unbalance the immune response to *Aspergillus* and favor the development of characteristic fungal infections. One such example is represented by cystic fibrosis (CF, OMIM#219700), an autosomal recessive disorder caused by mutations in the gene encoding for the Cystic Fibrosis transmembrane conductance regulator (*CFTR*, on chr. 7q31.2), a chloride/bicarbonate channel that regulates the electrolyte content of luminal fluid. *CFTR* mutations result in viscid secretion and defective airway mucociliary clearance providing a suitable environment for persistent microbial colonization (2). CF patients typically develop an extreme form of asthma, named allergic bronchopulmonary aspergillosis (ABPA), characterized by an exaggerated hypersensitivity reaction and associated with progressive lung function decline. More severe manifestations of *Aspergillus*-related diseases, such as IA, are rare in CF patients (3). In contrast, severe IA occur in severely immunocompromised patients such as those diagnosed with acute leukemia and chronic lymphoproliferative disorders, or recipients of allogeneic hematopoietic stem cell and solid-organ transplants (4).

The host immune response to *Aspergillus* has been extensively investigated and different cell types and signaling pathways have been brought into play (5). A central role at the *Aspergillus*/host interface is thought to be played by the enzyme indoleamine 2,3-dioxygenase 1 (IDO1) that, along with IDO2 and tryptophan 2,3-dioxygenase (TDO), catalyzes the first and rate-limiting step of tryptophan catabolism along the kynurenine pathway (6). IDO1 is expressed by several immune and non-immune cells and is recognized as a suppressor of inflammation and regulator of immune homeostasis, and failure to balance IDO1-mediated tolerance with the inflammatory response has been implicated in a wide range of diseases (7). Indeed, our group has recently shown that IDO1 enzyme was defective in murine CF and the decreased tryptophan/kynurenine metabolism was causally linked to *Aspergillus* infection via an unbalanced type 17 helper T cell/regulatory T cell response (8). Similarly, IDO1 has gained attention for its ability to control inflammation, pathogen immunity and tolerance in transplant recipients eventually leading to prevention of graft-vs-host reaction and reduction of aspergillosis incidence rates (9).

An additional level of complexity in the relationship between *Aspergillus* and the host is represented by the individual genetic background. Indeed, the presence of single-nucleotide polymorphisms (SNPs) that alter the expression and/or function of the molecules engaged in the cross-talk with *Aspergillus* may skew the balance between inflammation and tolerance and impact on the individual response to infection (9). Polymorphisms in the genes encoding for the enzymes involved in the kynurenine pathway, including IDO1, have been associated with several diseases (10). In the setting of fungal infection, our group has shown that *IDO1* rs3808606 T/T genotype correlated with a decreased susceptibility to recurrent vulvovaginal candidiasis and was associated with high levels of IL-22 and decreased levels of IL-17A and TNFα, enhanced IDO1 expression in vaginal cells and increased kynurenine-to-tryptophan ratio (11).

IDO biology has been recently enriched by the identification of a closely related protein, named IDO2 (12–14). *IDO1* and *IDO2* genes are adjacent to each other on chromosome 8p11.21 and likely arose by gene duplication before the divergence of vertebrates (15). In spite of the structural homology and the similar enzymatic activity, although with lower affinity, the function of IDO2 is still disputed, as well as how it coordinates with IDO1 activity (16).

Based on these premises, we have analyzed *IDO1*/*IDO2* polymorphisms in two different cohorts of patients, i.e., CF patients and recipients of hematopoietic stem cell transplantation (HSCT). We found that polymorphisms affecting the expression and/or function of IDO1 associate with increased risk of aspergillosis in the two cohorts of patients. In contrast, IDO2 appears to be dispensable in CF but required for optimal antifungal effector phagocytic activity in HSCT.

## Materials and Methods

### Ethics Statement

Murine experiments were carried out according to Italian Approved Animal Welfare Authorization 360/2015-PR and Legislative Decree 26/2014 regarding the animal license obtained by the Italian Ministry of Health lasting for 5 years (2015–2020).

The human study approval was provided by the University of Perugia ethics committee (Prot. 2012-043) and institutional review boards at each site. Written informed consent was obtained from all the participants, or, in case of minors, from parents or guardian.

### Cohort of CF Patients

Two hundred seventy-two Caucasian patients who had a proven diagnosis of CF (*CFTR* genotyping, sweat testing, and clinical phenotype) were enrolled in the study. Clinical records from each patient were reviewed and clinical data including age, gender, lung function testing, measures of nutrition, microbiological findings, and vital status were recorded (Table 1). *A. fumigatus* positivity was defined as the presence of persistent positive *Aspergillus* cultures, but negative galactomannan and no immunological responses or bacterial positivity as persistent, for at least 6 months. Study approval was obtained from institutional review boards at each site and written informed consent was obtained from the participants, or, in case of minors, from parents or guardian.

**Table 1:** Demographic and clinical characteristics of CF patients.

		***N***	**Mean ± S.D**.	**Range**
**Age in years:**		272	15.5 ± 11.4	0.1 - 56
**Age at diagnosis in months:**		255	31.8 ± 70.6	0 - 408
**FEV1, % predicted:**		204	84.8 ± 26.7	22 - 134
**FVC, % predicted:**		204	91.1 ± 21.7	29 - 131
**Height, cm:**		213	151.1 ± 24.6	71 - 192
**Weight, kg:**		213	48.7 ± 19.0	8 - 103
**BMI:**		211	20.2 ± 3.8	13 - 42
		***N***	**Percent**	
**Sex**:	Male	131	48.2%	
	Female	141	51.8%	
***CFTR*** **mutation**:	ΔF508 homozygous	72	26.8%	
	ΔF508 heterozygous	125	46.4%	
	Other	72	26.8%	
**Microbiological status**	**Status**	***N***	**Percent**	
*Aspergillus*	Yes	76	27.9%	
	No	196	72.1%	

*FEV1, forced expiratory volume in the first second; FVC, forced vital capacity; BMI, body mass index; CFTR, cystic fibrosis transmembrane conductance regulator. Continuous variables are expressed as mean ± SD*.

### Cohort of Hematological Patients Undergone HSCT

Three hundred fifty-two adult patients with hematologic disorders undergoing allogeneic HSCT at the transplantation center in Perugia (Italy) and their respective donors were enrolled in the study. Clinical records from each patient were reviewed and clinical data including age, gender, disease, transplantation type, GvHD and microbiological findings were recorded (Table 2). Seventy-eight cases of invasive aspergillosis were classified as “probable” or “proven,” according to the revised standard criteria from the European Organization for Research and Treatment of Cancer–Mycoses Study Group (17).

**Table 2:** Characteristics of HSCT recipients.

**Clinical variable**	**IA (*N* = 50)**	**Non-IA (*N* = 302)**	**RR**	**P**
**Age at transplantation–Mean ± S.D**.	37.3 (13.1)	40.3 (15.0)	0.988	0.130
**Females–*****n*** **(%)**	26 (52.0)	151 (50.0)	1.080	0.780
**HLA matching, no. (%)**				
Transplant from haploidentical- related donor	35 (70.0%)	146 (48.3%)	2.440	**0.003**
Transplant from half-matched related donor	4 (8.0%)	79 (26.2%)	0.261	**0.009**
Transplant from haploidentical unrelated donor	6 (12.0%)	51 (16.9%)	0.669	0.350
Transplant from half-matched unrelated donor	5 (10.0%)	26 (8.6%)	1.100	0.830
**Underlying disease, no. (%)**				
Acute leukemia (AML, ALL)	32 (64.0%)	231 (76.5%)	0.592	0.078
Lymphoma/myeloma (MM, HL, NHL-B)	13 (26.0%)	56 (18.5%)	1.440	0.248
Chronic leukemia (CML, CLL)	3 (6.0%)	14 (4.6%)	1.310	0.660
Other (SM, MDS, AA)	2 (4.0%)	1 (0.3%)	6.020	**0.003**
**Advanced disease stage, no. (%)**	29 (58.0%)	169 (56.0%)	1.100	0.720
**Myeloablative conditioning regimen, no. (%)**				
Total-body irradiation	42 (84.0%)	229 (75.8%)	1.620	0.213
No total-body irradiation	8 (16.0%)	73 (24.1%)	0.617	
**CMV serology of donor and recipient, no. (%)**				
CMV^−^/CMV^−^	5 (10.0%)	25 (8.3%)	1.056	0.900
CMV–/CMV+, CMV+/CMV– or CMV+/CMV+	45 (90.0%)	277 (91.7%)	0.947	
**GVHD, grade II to IV, no. (%)**	6 (12.0%)	21 (7.0%)	1.700	0.220

*P-values were calculated using competing risk logistic regression models. Statistically significant results are marked in bold. IA, invasive aspergillosis; HLA, human leukocyte antigen; TBI, total body irradiation; CMV, cytomegalovirus; GVHD, graft-vs-host-disease*.

### SNPs Selection and Genotyping

Four *IDO1* SNPs (Supplementary Table 1) were selected based on their ability to tag surrounding variants (*r*^2^ > 0.8, MAF > 0.05) in the HapMap-CEU population of the International HapMap project, phase III (18), using Haploview (19). Since *IDO2* gene was not annotated in the International HapMap project, phase III, we selected four tagSNPs based on literature review (20) and their location across *IDO2* coding region (Supplementary Table 1). Patients provided a blood specimen for DNA isolation performed using the QIAamp DNA Mini (Qiagen, Milan, Italy) following the manufacturer's instructions and stored at −20°C. SNP genotyping was performed using KASPar assays (LGC Genomics) according to manufacturer's instructions in an Applied Biosystems 7500 Fast qPCR system (Life Technologies). Genotyping sets comprised randomly selected replicates of previously typed samples and two negative controls (water). Concordant genotyping was obtained for ≥99%. Hardy-Weinberg Equilibrium (HWE), Minor Allele Frequency (MAF) and genotyping rate were determined using Haploview (19). SNPs with a genotyping rate < 90% were not included in the genetic association testing. No HWE cut-off was applied since both cohorts were composed by affected subjects, and in this situation a deviation from HWE may be indicative of causative effect at the considered loci.

### Genetic Association Testing

The CF and HSCT datasets have been designed as case-control and longitudinal study, respectively, and the analysis of their genetic data has been performed using different approaches.

For the CF dataset, haplotype and single-SNP analyses have been performed using PLINK (21), using logistic or linear regression, adjusting for sex and age-at-sampling. The association testing in the HSCT cohort was performed by determining the cumulative incidence of IA at 24 months after transplantation applying competing risk logistic regression models, adjusting for *HLA* matching and underlying disease, considering transplant related mortality and relapse as competing risks. Competing risk logistic regression models were tested according to donor/recipient status using R software (22).

Statistical significance was set up according to the different hypotheses tested throughout the work. For the primary aim of the study, that is testing the impact of *IDO1*/*IDO2* loci on *Aspergillus* infection in CF patients, we set up a *P* < 0.025 (0.05/2) for haplotype and *P* < 0.006 (0.05/8) for single-SNP analysis according to Bonferroni's correction [2 Linkage Disequilibrium (LD) blocks for haplotype omnibus test and 8 independent SNPs]. LD blocks were defined using Haploview (19) according to the solid spine algorithm. For the association testing in the HSCT cohort we considered a nominal *P* < 0.01 (0.05/5) as statistical significance threshold given the number of SNPs tested (*N* = 5). All the single-SNP analyses have been performed according to three genetic models of association (additive, dominant and recessive). No Bonferroni's correction has been applied according to the three genetic models of association since they do not represent a mode of independent testing.

### Mice

Female, 8- to 10-weeks-old C57BL6 and Balb/c mice were obtained from Charles River Laboratories (Calco, Italy). B6129indo (*Ido1*^−/−^) mice were bred under specific pathogen-free conditions in the animal facility at the University of Perugia.

### *Aspergillus fumigatus* Strain and Infection

Viable conidia (95%) of *A. fumigatus* (Af293) were obtained by growth on Sabouraud dextrose agar (Sigma-Aldrich) supplemented with chloramphenicol for 5 days at room temperature. The fungus was collected with a cell scraper after addition of PBS, transferred in a tube, pelleted and resuspended in PBS. After counting, the resuspension was further diluted in PBS to reach the desidered final concentration. For *in vivo* experiments, mice were anesthetized by intraperitoneal injection of 2.5% avertin (Sigma Chemical Co, St. Louis, MO) before intranasal instillation of a suspension of 2 × 10^7^ resting conidia /20 μl saline. For *in vitro* experiments, cells were treated at a 1:20 cell/conidia ratio.

### Bone Marrow (BM) Transplantation Model and Infection

BM cells from donor mice were prepared by differential agglutination with soybean agglutinin. T cell-depleted cells (1 × 10^7^ containing 1% of contaminating T cells on FACS analysis) were injected intravenously into recipient Balb/c mice exposed to a lethal radiation dose of 9 Gy. 95% of the mice survived, showing a stable, donor type haematopoietic chimaerism, as revealed by donor type major histocompatibility (MHC) class I antigen expression on cells from spleens, as previously described (23). BM cells from *Ido2*^−/^^−^ mice were kindly provided by G.C. Prendergast (Lankenau Institute for Medical Research (LIMR), USA). Mice were monitored for 4 days for fungal growth which was expressed as log_10_ CFU per organ, mean±SD.

### Histological Analysis

Lungs were removed and immediately fixed in 10% neutral buffered formalin (Bio-optica, Milan) for 24 h, embedded in paraffin, sectioned into 3–4 μm and stained with Periodic Acid-Schiff reagent. Histology images were acquired using a microscope (BX51 Olympus) with a 40× objective equipped with a high-resolution DP71 camera (Olympus).

### Isolation of Splenic Macrophages and Conidiocidal Activity

Adherent spleen cells were isolated after treating the whole organ with the injection of 2 mL of Collagenase D (Sigma) at 2 mg/mL. The spleen tissue was cut into small pieces and incubated for 20 min at 37°C in the enzyme solution. The cell suspension and remaining tissue fragments were suspended in culture medium and gently passed using a syringe plug through a 100 μm cell strainer to obtain a single cell suspension. Cells were cultured in a 75 cm^2^ flasks and were allowed to adhere for 4 h at 37°C and the non-adherent cells were removed after washing the monolayers with pre-warmed PBS. Five hundred thousand adherent cells (macrophages) were then incubated at 37°C with resting *Aspergillus* conidia (fungi/cell ratio 1:3) for 120 min in 96-well flat-bottom microtiter plates and the percentage of CFU inhibition (mean), referred to as conidiocidal activity, was determined as follows: 100–(CFU in experimental group/CFU in control cultures) × 100.

### Human Bronchial Epithelial Cells and Human PBMC Culture and Treatment

Human bronchial epithelial (HBE) cells were obtained from lung transplants (CF patients homozygous for the *CFTR* ΔF508 mutation) or lung resections (non-CF patients) and cultured as described (24). PBMC fractions were obtained by density centrifugation of diluted blood (one part blood to one part pyrogen-free saline) over Ficoll-Paque (Pharmacia Biotech; Uppsala). PBMCs were washed twice in saline and suspended in culture medium supplemented with gentamicin 1%, L-glutamine 1%, and pyruvate 1%. The cells were counted in a Bürker counting chamber, and their number was adjusted to 5 × 10^6^ cells/ml. Five hundred thousand PBMCs in a volume of 200 μl per well were incubated at 37°C in round-bottom 96-well, in the presence of 10% human pooled serum, with stimuli or culture medium alone. Cells were treated for 24 h with either IFNγ (200 U/ml) or *A. fumigatus* conidia (1:20 cell/conidia ratio). In the latter condition, amphotericin B was added after 6 h to prevent hyphal formation.

### Immunofluorescence Staining

Monocytes from healthy donors were exposed to live *A. fumigatus* conidia. After washout, cells were fixed in 2% formaldehyde for 40 min at room temperature and permeabilized in a blocking buffer containing 5% FBS, 3% BSA, and 0.5% Triton X-100 in PBS. The cells were then incubated overnight at 4°C with primary antibody against IDO1 protein (IDO antibody LS-C153780) in a buffer containing 3% BSA and 0.1% Triton X-100 in PBS. After extensive washing with PBS, the cells were incubated at room temperature for 60 min with 1:400 secondary anti-mouse IgG–TRITC antibody (Sigma-Aldrich). Alexa Fluor® 488 phalloidin was used for selective labeling of F-actin and nuclei were counterstained with DAPI. Images were acquired using a fluorescence microscope (BX51, Olympus) with a 100 × objective and analySIS image processing software (Olympus).

### Western Blotting

Cells were lysed in 2x Laemmli buffer (Sigma-Aldrich). Blots of cell lysates were incubated with an antibody against IDO1 (clone 10.1, Millipore) followed by IgG–HRP-conjugated secondary antibody (Sigma–Aldrich) after separation in 10 or 12% Tris/glycine SDS gel and transferred to a nitrocellulose membrane. A cell lysate of IFNγ-stimulated HeLa cells was used as positive control in selected experiments for correct assignment of IDO1 band. Normalization was performed probing the membrane with mouse-anti-β-tubulin antibody (Sigma–Aldrich, clone T9026). Chemiluminescence detection was performed with LiteAblotPlus chemiluminescence substrate (Euroclone S.p.A), using the ChemiDocTM XRS+Imaging system (Bio-Rad), and quantification was obtained by densitometry image analysis using Image Lab 6.0 software (Bio-Rad).

### Real-Time PCR

Real-time PCR was performed using the iCycler iQ detection system (Bio-Rad) and iTaq™ Universal SYBR® Green Supermix (Biorad). Total RNA was extracted using RNeasy Mini Kit (QIAGEN, Milan, Italy) and reverse transcribed with Sensiscript Reverse Transcriptase (QIAGEN) according to the manufacturer's directions. Amplification efficiencies were validated and normalized against β-actin. The thermal profile for SYBR Green real-time PCR was at 95°C for 3 min, followed by 40 cycles of denaturation for 30 s at 95°C and an annealing/extension step of 30 s at 60°C. Each data point was examined for integrity by analysis of the amplification plot. The following primers were used: human IDO1: forward TCACAGACCACAAGTCACAG, reverse GCAAGACCTTACGGACATCT; human ACTB: forward CACTCTTCCAGCCTTCCTTCC, reverse ACAGCACTGTGTTGGCGTAC; mouse *Tnf* : forward CGAGTGACAAGCCTGTAGCC, reverse AAGAGAACCTGGGAGTAGACAAG; mouse *Actb*: forward AGCCATGTACGTAGCCATCC, reverse CTCTCAGCTGTGGTGGTGAA.

### ELISA

Human IL-6, IL-8, and IL-17A cytokine concentration was determined in HBE supernatants or in BAL from HSCT patients by using specific ELISA kits according to manufacturers' instructions (eBioscience Inc., R&D System and Biolegend).

### Kynurenine and Tryptophan Assay

IDO functional activity was measured *in vitro* in terms of the ability to metabolize tryptophan to kynurenine whose concentrations were measured by high-performance liquid chromatography (25).

### Statistical Analysis

One-way ANOVA with Bonferroni *post-hoc* test was used to determine the statistical significance. Significance was defined as *p* < 0.05. Data are pooled results (mean ± SD) or representative images from three experiments. GraphPad Prism software 6.01 (GraphPad Software) was used for analysis.

## Results

### Genetic Variability at *IDO1* Locus Associates With *Aspergillus* Infection in CF Patients

**Figure 1:**
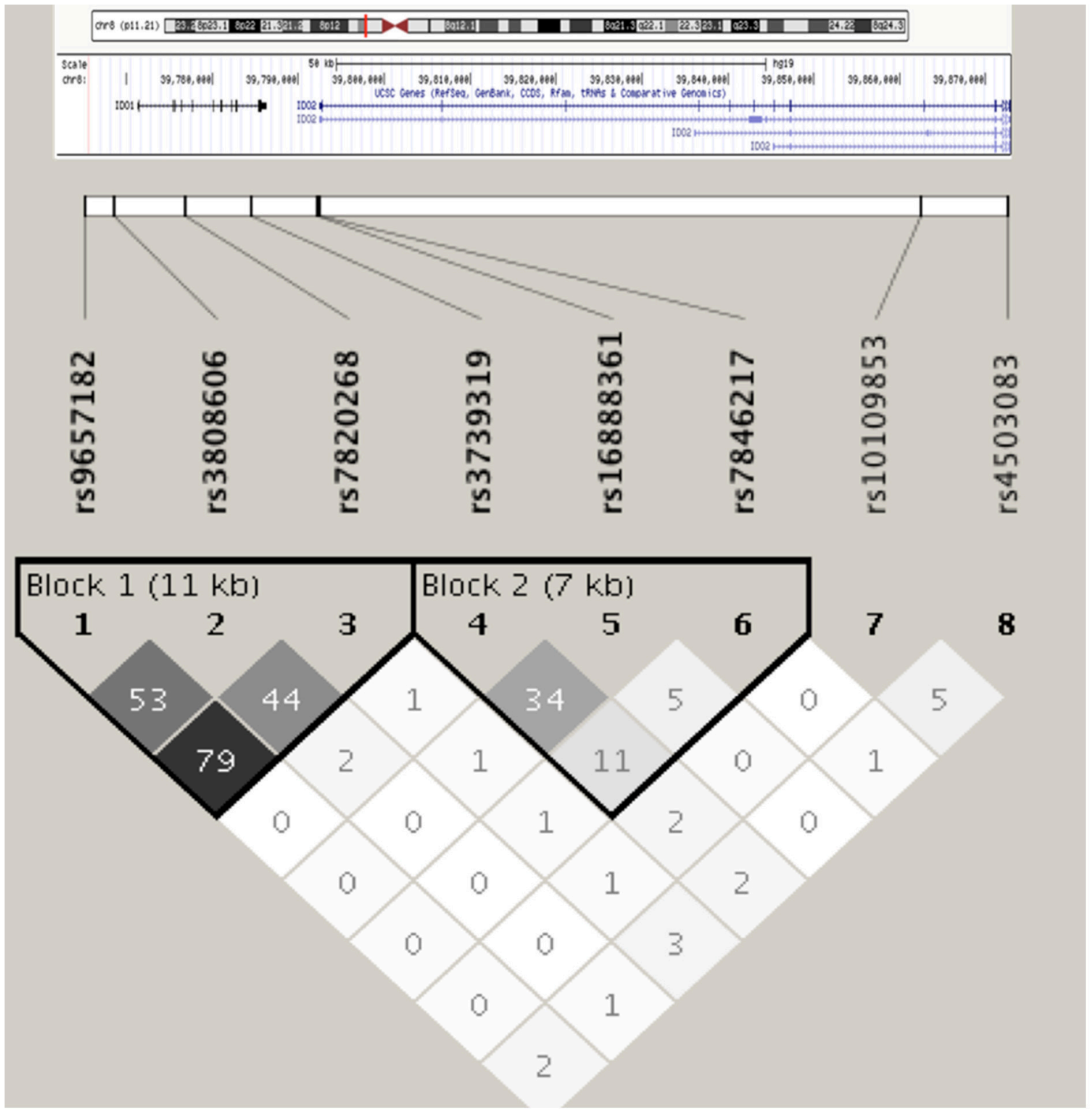
Linkage disequilibrium among selected *IDO1*/*IDO2* tagSNPs in the cohort of CF patients. Haplotype blocks are defined according to the solid spine of LD algorithm. Linkage disequilibrium is expressed in r2.

The eight tagSNPs encompassing *IDO1*/*IDO2* loci have been successfully genotyped in the CF cohort with genotyping rate above 90%, not showing significant deviation from HWE and having MAFs comparable to the ones reported for the European population of the 1,000 Genomes Consortium (26) (Supplementary Table 1). Linkage disequilibrium (LD) analysis of the tagSNPs revealed the existence of two LD blocks of 11 and 7 kb, respectively, with the first one spanning from the 5′-end to the 4th intron of *IDO1* locus, and the second one ranging from the last intron of *IDO1* to the first intron of *IDO2* (Figure 1).

Haplotype analysis of the two LD blocks showed a significant association of the first LD block with *Aspergillus* infection (omnibus-*P* = 0.001), while the testing of the second LD block did not turn significant (omnibus-*P* = 0.137). In particular, haplotype 3 (H3) and 4 (H4) at the first LD block displayed significant association with reduced (H3, OR = 0.278, *P* = 0.001) and increased (H4, OR = 2.710, *P* = 0.008) risk of *Aspergillu*s infection (Table 3).

**Table 3:** Haplotype association test in the cohort of CF patients.

**LD block**	**Haplotype**	**rs9657182**	**rs3808606**	**rs7820268**	**Freq cases**	**Freq controls**	**OR**	**P**
1	1	C	T	C	49.7%	41.1%	1.410	0.089
	2	T	C	T	34.1%	37.1%	0.859	0.487
	3	C	C	C	5.5%	17.8%	0.278	**0.001**
	4	T	C	C	9.8%	3.3%	2.710	**0.008**
**LD block**	**Haplotype**	**rs3739319**	**rs16888361**	**rs7846217**	**Freq cases**	**Freq controls**	**OR**	**P**
2	1	G	G	T	34.5%	45.0%	0.640	0.041
	2	A	A	T	24.1%	21.9%	1.190	0.460
	3	A	G	T	20.7%	18.8%	0.970	0.906
	4	G	G	C	18.4%	12.9%	1.550	0.112
	5	G	A	T	2.2%	1.4%	3.120	0.164

*LD blocks were defined using Haploview (19) according to the solid spine algorithm. Haplotype association tests were performed using PLINK (21), adjusting for age-at-sampling and sex. Significant results are highlighted in bold*.

Single SNP analysis of the eight tagSNPs across the two loci reported the nominal association of *IDO1* rs3808606 (additive OR = 1.513, *P* = 0.044; recessive OR = 2.283, *P* = 0.017), *IDO1* rs9657182 (recessive OR = 2.236, *P* = 0.024) and *IDO2* rs7846217 (dominant OR = 1.989, *P* = 0.037) with increased risk of *Aspergillus* infection (Table 4).

**Table 4:** Single-SNP association test in the cohort of CF patients.

**SNP**	**ALLELE**	**N**	**MODEL**	**OR**	**P**	**MODEL**	**OR**	**P**	**MODEL**	**OR**	**P**
rs9657182	T	270	ADD	1.186	0.408	DOM	0.825	0.529	**REC**	**2.236**	**0.024**
rs3808606	T	264	**ADD**	**1.513**	**0.045**	DOM	1.388	0.310	**REC**	**2.283**	**0.017**
rs7820268	T	271	ADD	0.857	0.479	DOM	0.743	0.311	REC	1.032	0.944
rs3739319	A	267	ADD	1.108	0.612	DOM	0.955	0.882	REC	1.462	0.279
rs16888361	A	263	ADD	1.307	0.253	DOM	1.319	0.349	REC	1.744	0.324
rs7846217	C	251	ADD	1.590	0.093	**DOM**	**1.989**	**0.037**	REC	0.901	0.899
rs10109853	T	248	ADD	0.995	0.982	DOM	0.890	0.730	REC	1.137	0.731
rs4503083	A	250	ADD	1.060	0.798	DOM	0.997	0.992	REC	1.368	0.541

*Genotype association tests were performed by logistic regression using PLINK (21), adjusting for age-at-sampling and sex. Nominally significant results are highlighted in bold. ADD, Additive; DOM, Dominant; REC, Recessive*.

### *IDO1* rs3808606 and *IDO2* rs7846217 Influence Immune Responses to *A. fumigatus in vitro*

*IDO1* rs3808606, located on the first LD block, showed the most consistent association signal in the single-SNP analysis and it is a whole-blood eQTL for *IDO1* according to Haploreg v4.1 (27). *IDO2* rs7846217, despite being located in the first intron of *IDO2* and in the second LD block in our analysis, is in LD with putative regulatory sites located toward the 3′-end of *IDO1* (Intron 9-Exon10-3′UTR) and a whole-blood eQTL for *IDO1* according to Haploreg v4.1 (27). Therefore, to gain mechanistic insights for the association of genetic variability at the *IDO1* locus with *Aspergillus* infection, we performed functional assays according to *IDO1* rs3808606 and *IDO2* rs7846217 SNPs.

**Figure 2:**
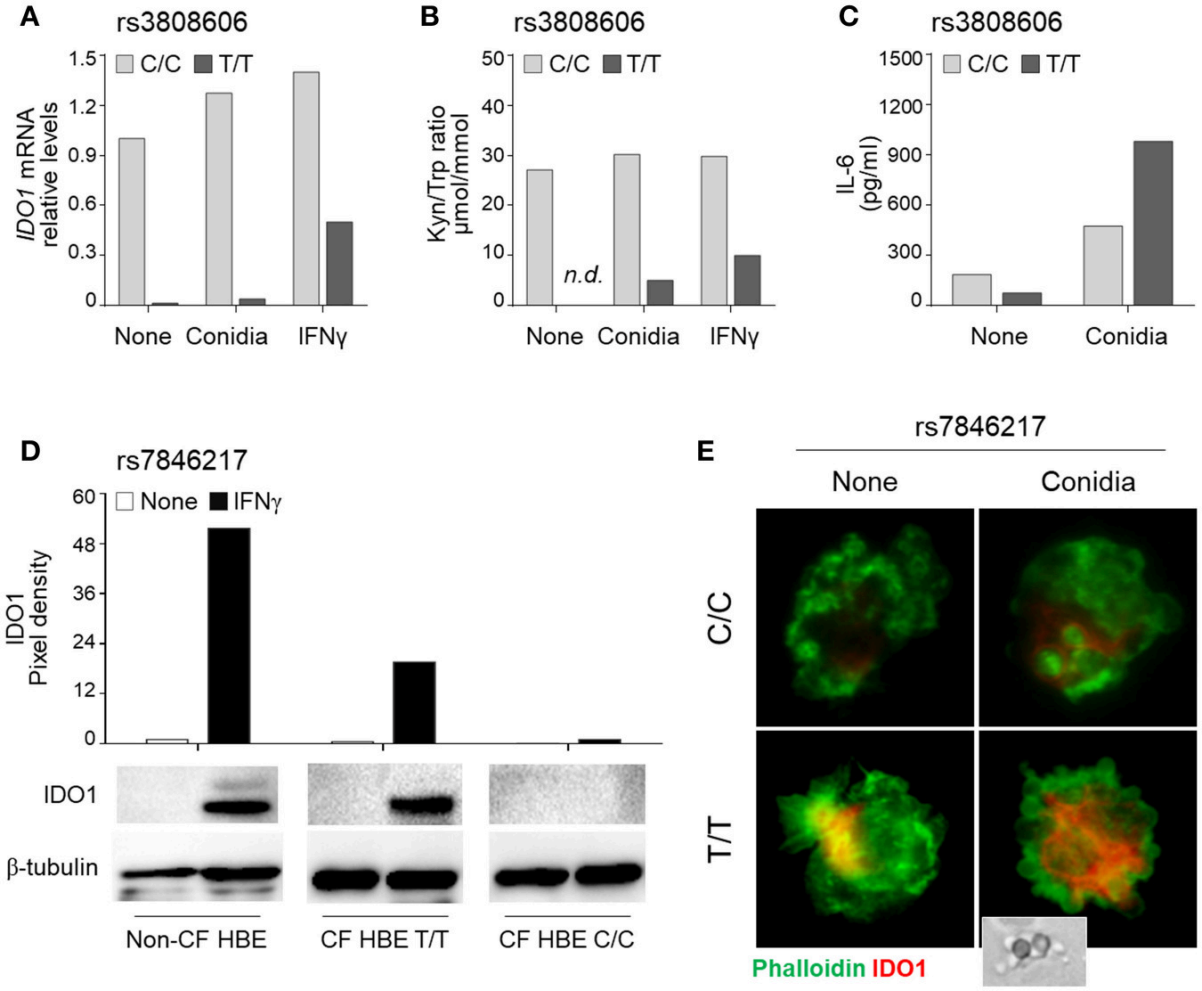
*IDO1* rs3808606 and *IDO2* rs7846217 impair IDO1 induction *in vitro*. HBE cells from CF patients carrying the two homozygous variants at rs3808606 (C/C, light gray bars; T/T, dark gray bars) **(A–C)** or rs7846217 (T/T, major allele; C/C, minor allele) **(D,E)** were treated with *A. fumigatus* conidia **(A–C,E)** or IFNγ **(A,B,D)**. Cell lysates were evaluated for IDO1 mRNA expression by RT-PCR **(A)** or protein levels by Western blot **(D)**. mRNA levels are expressed as relative levels of unstimulated (none) HBE cells from the CF patient carrying the major allele. Protein values are expressed as relative levels of unstimulated (none) HBE cells from the non-CF patient. Supernatants were analyzed for kynurenines and tryptophan levels by high-performance liquid chromatography **(B)** and for IL-6 levels by ELISA **(C)**. Monocytes from healthy donors carrying the two homozygous variants at rs7846217 (T/T, major allele; C/C, minor allele) were treated with *A. fumigatus* conidia (in the inset) and evaluated by immunofluorescence for the expression of IDO1 (red). Phalloidin was used to stain actin filaments (green) **(E)**. Shown is a representative experiment from three independent experiments.

We first evaluated the expression and function of IDO1 in HBE cells from CF patients according to their rs3808606 genotype. IDO1 mRNA levels were lower in T/T carriers as compared to the C/C genotype (Figure 2A). The stimulation with *Aspergillus* conidia or IFNγ induced *IDO1* mRNA in HBE cells carrying the C/C genotype, and in lower amount, in HBE cells carrying the T/T genotype (Figure 2A). Quantification of the kynurenine/tryptophan ratio demonstrated that resting HBE cells bearing the T/T genotype produced levels of kynurenines that were below the threshold of detection and remained low even in the presence of *Aspergillus* conidia or IFNγ (Figure 2B). These results demonstrate that HBE cells from CF patients bearing the T/T genotype at rs3808606 display defective mRNA expression and enzymatic activity of IDO1, both in resting conditions and upon stimulation with conidia. Further confirming the impaired IDO1 activity, the levels of IL-6 were higher in HBE cells bearing the T/T genotype as compared to those bearing the C/C genotype (Figure 2C).

Consistent with *IDO2* rs7846217 being a whole-blood eQTL for *IDO1*, HBE cells showed impaired IDO1 expression upon stimulation with IFNγ when bearing the C/C, but not the T/T, genotype (Figure 2D). Similarly, human monocytes stimulated with *Aspergillus* conidia showed reduced expression of IDO1 when bearing the C/C, but not the T/T, genotype (Figure 2E).

Interestingly, the two non-synonymous *IDO2* SNPs, rs10109853 (NP_919270.2:p.Arg248Trp), and rs4503083 (NP_919270.2:p.Tyr359Ter), which profoundly impair IDO2 activity, were not associated with *A. fumigatus* infection in CF patients (Table 4).

Overall, the genetic association study in the cohort of CF patients would suggest, and confirm, the key role played by IDO1 in response to the fungus, as two distinct SNPs, located within and downstream the *IDO1* gene, that impair IDO1 function along with an increased inflammatory response, are risk factors for aspergillosis in these patients. At variance, IDO2 appears dispensable as SNPs that profoundly affect its expression or function do not associate with an increased risk.

### *IDO1* rs7820268 and *IDO2* rs10109853 and rs4503083 Associate With *Aspergillus* Infection in HSCT Patients

Based on the results obtained in the CF cohort, we extended our analyses of *IDO1*/*IDO2* polymorphisms to the cohort of HSCT patients. Five of the eight tagSNPs encompassing *IDO1*/*IDO2* loci have been successfully genotyped in the HSCT cohort with genotyping rate above 90% (Supplementary Table 1). The MAF at the five tagSNPs is comparable to the ones reported for the European population of the 1,000 Genomes Consortium (26) (Supplementary Table 1). No significant differences in MAF were detected between CF and HSCT patients, and no linkage disequilibrium was detected among the 5 tagSNPs analyzed. However, both *IDO2* rs10109853 and rs4503083 showed significant deviation from HWE (Supplementary Table 1). In particular, the genotype distribution of rs4503083 deviates from HWE only in recipients (*P* = 0.0007), suggesting a possible predisposing effect to the underlying pathology. Conversely, when separating recipients from donors according to rs10109853, no significant deviation from HWE was detected. The analysis of clinical/demographical characteristics in transplant recipients showed that both *HLA* matching and underlying disease status significantly affected the risk of *Aspergillus* infection (Table 2). Thus, we considered these two variables as covariates in the logistic regression models testing the association of *IDO1*/*IDO2* SNPs with *Aspergillus* infection. Genetic association results are reported in Table 5. *IDO1* rs7820268 showed a significant association in transplant recipients, with minor allele T increasing additively the risk of *Aspergillus* infection (RR = 1.685, *P* = 0.009). Moreover, the two *IDO2* SNPs affecting protein coding sequence, rs10109853 and rs4503083, double the risk of *A. fumigatus* infection when present in the HSCT donors in a genetic recessive model (rs10109853, RR = 2.214, *P* = 0.012; rs4503083, RR = 2.841, *P* = 0.015, Table 5).

**Table 5:** Genetic association testing in the BMT cohort.

			**Additive**		**Dominant**		**Recessive**	
**SNP**	**Allele**	***N***	**RR**	***P***	**RR**	***P***	**RR**	***P***
**RECIPIENTS**								
rs782026	T	293	1.685	0.009	*2.350*	*0.016*	1.535	0.360
rs16888361	A	300	1.416	0.140	1.590	0.150	1.462	0.460
rs7846217	C	303	0.921	0.790	0.845	0.650	1.176	0.820
rs10109853	T	293	1.109	0.620	1.342	0.430	0.982	0.960
rs4503083	A	304	1.004	0.990	0.979	0.950	1.095	0.890
**DONORS**								
rs782026	T	325	0.777	0.210	0.986	0.960	0.152	0.066
rs16888361	A	32	0.932	0.770	0.923	0.780	0.900	0.860
rs7846217	C	320	0.984	0.960	0.957	0.900	1.250	0.840
rs10109853	T	314	1.217	0.400	0.789	0.430	*2.214*	*0.012*
rs4503083	A	322	0.996	0.990	0.730	0.320	*2.841*	*0.015*

*P-values and relative risk (RR) were calculated using competing risk logistic regression models, adjusting for HLA matching and underlying disease, considering transplant related mortality and relapse as competing risks. Statistically significant results are marked in bold. Nominally-significant results are highlighted in Italics*.

### *IDO1* rs7820268 SNP Influences IDO1 Function

**Figure 3:**
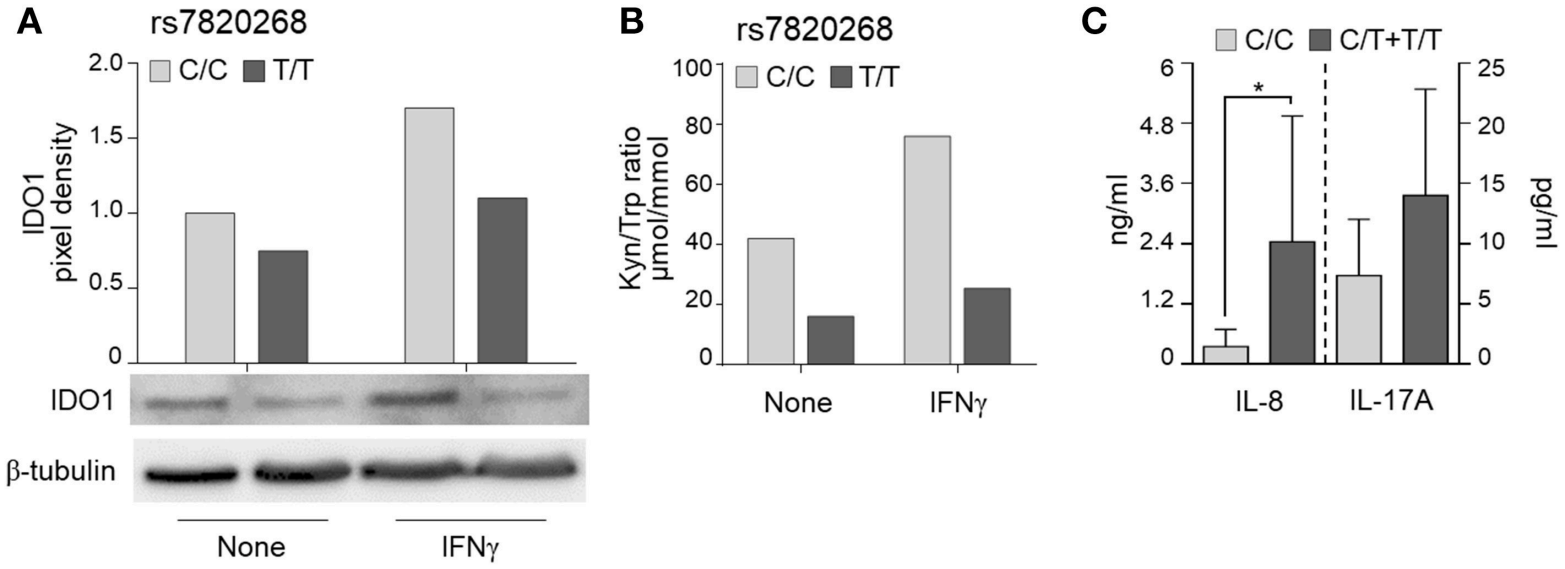
*IDO1* rs7820268 associates with defective IDO1 expression and enzymatic activity. (A,B) PBMC from donors carrying the two homozygous variants at rs7820268 (C/C, light gray bars; T/T, dark gray bars) were treated with IFNγ. Cell lysates were evaluated for IDO1 protein expression by Western blot (A). Protein values are expressed as relative levels of unstimulated (none) cells from donors carrying the major allele. Supernatants were analyzed for kynurenines and tryptophan levels by high-performance liquid chromatography (B). Shown is a representative experiment from three independent experiments. (C) BAL from HSCT patients with suspected infection carrying the homozygous or heterozygous variants at rs7820268 (C/C, light gray bars; C/T + T/T, dark gray bars) were evaluated for IL-8 and IL-17A levels by ELISA (*n* = 7). Data are expressed as mean ± SD. ^*^p < 0.05.

To investigate whether the *IDO1* rs7820268 polymorphism was associated with impaired enzyme expression and activity, we first measured IDO1 protein levels in PBMC from healthy controls and found a reduced expression in both resting and IFNγ-stimulated PBMC bearing the T/T genotype (Figure 3A). We also measured the kynurenine/tryptophan (Kyn/Trp) ratio in the supernatants of PBMC left untreated or stimulated with IFNγ and found decreased levels among carriers of T/T compared with C/C genotypes, indicating a reduced enzymatic activity (Figure 3B). These results are in agreement with published data (28).

Consistent with the notion that inflammatory responses are increased in conditions of impaired IDO1 function (9), the levels of cytokines in the bronchoalveolar lavage (BAL) fluids of patients with *Aspergillus* showed significant differences according to *IDO1* rs7820268 genotype, being Th17 (IL-8 and IL-17A) higher in C/T+T/T carriers compared to C/C patients (Figure 3C). Together, these results suggest that impaired IDO1 function along with increased inflammatory response are risk factors for aspergillosis in HSCT patients similarly to what observed in CF patients.

### IDO2 in Hematopoietic Cells Protects Against Aspergillosis in a Murine Model of HSCT

**Figure 4:**
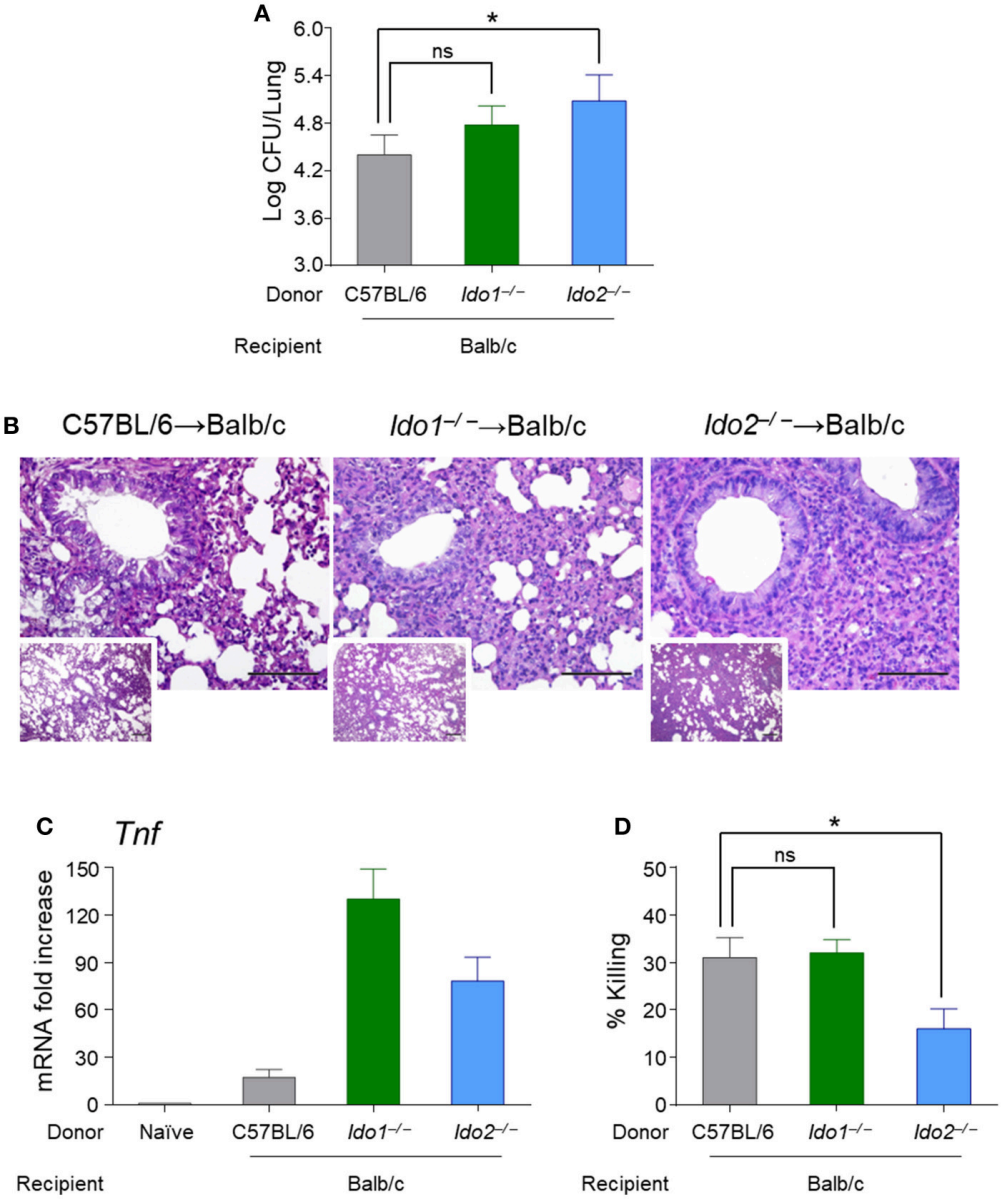
Mice transplanted with bone marrow-derived cells from *Ido2*^−/−^ mice have a more severe pathology upon challenge with *A. fumigatus*. Balb/c mice were transplanted with bone marrow derived cells from wild-type, *Ido1*^−/−^ or *Ido2*^−/−^ mice and challenged with *A. fumigatus* for 4 days. Mice were assessed for fungal burden in the lung (log10 CFUs) (A), histology (PAS; the scale bar represents 500 or 100 μm) (B), *Tnf* expression (RT-PCR) (C), and antifungal effector activity by splenic macrophages (% killing) (D). Data represent pooled results (*n* = 3, mean ± SD) or representative images from three experiments. ^*^p < 0.05; ns, not significant.

The finding that the *IDO2* SNPs displayed nominal associations with *A. fumigatus* infection when present in the HSCT donors prompted us to study the potential role of IDO2 in hematopoietic transplantation. To this purpose, we resorted to Balb/c mice transplanted with bone marrow derived cells from *Ido1*^−/−^ or *Ido2*^−/−^ (kindly donated by G.C. Prendergast (Lankenau Institute for Medical Research (LIMR), USA) mice, before challenge with *A. fumigatus*. Mice were assessed for fungal burden in the lung, parameters of inflammatory pathology and antifungal effector activity. We found an increased local fungal burden in *Ido1*^−/−^ or *Ido2*^−/−^ reconstituted mice (Figure 4A) that was associated with a more severe immunopathology, particularly in *Ido2*^−/−^-reconstituted mice (Figure 4B), and an increased *Tnf* expression (Figure 4C). Of interest, a significant reduction of conidiocidal activity in splenic macrophages was also observed in *Ido2*^−/−^-reconstituted mice (Figure 4D). Collectively, these results suggest that IDO2 may play an unanticipated role in *A. fumigatus* infection by sustaining the phagocytic antifungal effector activity.

## Discussion

The results presented in this work extend our current knowledge on the role of IDOs in the host response to *Aspergillus*. By performing genetic association studies in CF patients and in recipients of HSCT, we confirmed the pivotal role of IDO1 in balancing immunity and tolerance to the fungus in the two clinical settings. It is known that IDO1 is defective in CF patients (8) and that it plays a role in aspergillosis in HSCT (9). Therefore, it is not surprising that SNPs reducing the expression and function of IDO1 are associated with aspergillosis in CF and HSCT patients, although further studies are required to establish how the single SNPs, alone or in combination, modulate IDO1 expression and function in the two cohorts of patients.

With regard to IDO2, our study showed that the two *IDO2* SNPs, leading to truncated or catalytically impaired IDO2 protein, do not associate with an increased risk for aspergillosis in CF patients. However, the same two *IDO2* SNPs associated with aspergillosis in HSCT recipients. Since IDO2 was cloned by three independent groups (12–14), deciphering the function of IDO2 has remained enigmatic until the generation of *IDO2* knock-out mice (29), when evidence of specific roles has begun to emerge. A substantial body of evidence has prompted the concept that IDO2 might act as a pro-inflammatory mediator in autoimmune diseases, specifically autoimmune arthritis (30–32), systemic lupus erythematosus (33) and in contact hypersensitivity (29). In these pathological settings, the role of IDO2 is B cell-intrinsic and likely affects the cross-talk between B and T cells (33). Interestingly, the clinical manifestation of aspergillosis in CF is in the form of allergic bronchopulmonary aspergillosis (3). Although type I (IgE-mediated) hypersensitivity is common, type III (IgG-mediated immune complex) and type IV (cell-mediated) reactions have also been observed (34). Based on this evidence, it is tempting to speculate that IDO2 might have a pro-inflammatory role also in ABPA. On this regard, a preliminary analysis testing the association between the eight tagSNPs with IgE levels in CF patients yielded a significant association of *IDO2* rs16888361, with minor allele A carriers having higher IgE levels compared to G/G carriers (β = 0.548, *P* = 0.005) (VN-personal communication). The precise effect of rs16888361 on IDO2 expression is currently under investigation. Interestingly, a pro-inflammatory role of IDO2 has been recently suggested in a study evaluating the influence of *IDO2* gene status in tumor progression and radiotherapy response in pancreatic ductal adenocarcinoma (PDAC) (35). In particular, an IDO2 deficient status, while being significantly absent in females with PDAC, was associated with an improved immune signature, further supporting the hypothesis that IDO2 may promote inflammatory responses, and this was associated with an improved survival in response to adjuvant radiotherapy (35).

If overreacting immune responses occur in CF patients in response to *Aspergillus*, HSCT patients are severely immunocompromised and fail to properly counteract *Aspergillus* infectivity. The kinetics and characteristics of the reconstitution of the immune system after HSCT provides useful hints to understand the susceptibility to infection (36). For instance, while the absolute number of neutrophils was normalized after 30 days, ROS production was critically impaired in patients with IA, and PMN-mediated killing of *A. fumigatus* remained significantly reduced over 1 year in all HSCT recipients (36). By using a murine model of HSCT, reconstitution of wild-type mice with *IDO2*-deficient hematopoietic stem cells resulted in a reduced conidiocidal activity of phagocytes, an event that could favor *Aspergillus* dissemination. While IDO2 expression and function have been consistently reported in B cells (29), in DCs (37) and also in macrophages (29), to the best of our knowledge it is unclear whether neutrophils express IDO2 and, more importantly, how it correlates to their conidiocidal activity. Studies are ongoing to address this issue.

Based on these observations, our results showing that IDO2 plays a context-dependent effect could be reconciled by taking into account the different environment in which the cross-talk between *Aspergillus* and the host takes place. Specifically, the hypersensitivity response in the dysregulated immune system in CF patients, in which IDO2 has a pro-inflammatory role, and the inadequate phagocytic response in patients undergone HSCT, in which IDO2 appears to be required for optimal conidiocidal activity, might account for the distinct association patterns of *IDO2* SNPs with aspergillosis in the two cohorts of patients.

Besides the specific role of IDO2, the interplay between IDO1 and IDO2 is also an active field of investigation. While the initial characterization of IDO2 was suggestive of a low capacity to produce kynurenine, the recent optimization of the requirements for proper evaluation of IDO2 enzymatic activity has demonstrated a clear ability to catabolize Trp (38). Therefore, IDO1 and IDO2 work in the same Trp-catabolic pathway and competition for the same substrate might occur. In addition, it has been suggested that IDO2 might negatively regulate IDO1 by means of its heme-binding site (39). Another possible interplay between IDO1 and IDO2, besides the enzymatic activity, might be the regulation of their reciprocal expression. For instance, it has been observed that both IDO1 and IDO2 are expressed in the epididymis and that the expression of IDO2 is strongly upregulated in conditions of IDO1 deficiency, although the increased expression was not sufficient to compensate for the reduced levels of kynurenines (40). A recent characterization of *Ido1* knockout mice has shown a defective IDO2 splicing and function, resulting in a mosaic knock-out for IDO2, thus suggesting the existence of an IDO1-IDO2 genetic interaction (29). Other studies have reported a down-regulation of the *Ido2* transcript in *Ido1* knock-out mice, which could be linked to loss of *Ido2* regulatory elements within the *Ido1* gene (41). For instance, *Ido2* mRNA was decreased in naïve as well as diseased inguinal lymph nodes from *Ido1* knock-out mice in a model of collagen-induced arthritis (42), and a 2-fold decrease was also reported in the liver, but not spleen (30). How and whether IDO1 and IDO2 cross-regulate in the overall response to the fungus remain to be investigated.

In conclusion, our results confirm and extend the role of IDO1 in the response to *Aspergillus*, and identifies IDO2 as an additional player. The development of selective IDO2 inhibitors, such as tenatoprazole (43), chloroquine (44) or indoximod (45), might help to differentiate between the distinct contributions of IDO1 and IDO2 without the confounding effects of cross-regulation at the genetic level. Alternative approaches may include the use of SNP knock-in mice to recapitulate the functional alterations observed in humans, a strategy already employed in different contexts to mimic human phenotypes (46–48). Whatever the approach, these results are expected to pave the way for the pharmacological targeting of IDO2 in aspergillosis in high-risk patients, as recently explored in autoimmune arthritis with the development of an IDO2-targeted therapeutic antibody (32).

## Data Availability

All datasets generated for this study are included in the manuscript and/or the Supplementary Files.

## Ethics Statement

All subjects gave written informed consent in accordance with the Declaration of Helsinki. The protocol was approved by the Institutional Review Board at each site.

This study was carried out according to Italian Approved Animal Welfare Authorization 360/2015-PR and Legislative Decree 26/2014 regarding the animal license obtained by the Italian Ministry of Health lasting for 5 years.

## Author Contributions

MP, MB, VO, CG, ADL, CS, CV, and GR designed and performed the experiments. VL, CaC, EF, CL-F, AC, LDA, FM, MCR, HE, AS, FA, and AV enrolled patients and collected clinical data and samples. VN, PM, SB, LR, and ClC analyzed the data and wrote the paper.

### Conflict of Interest Statement

The authors declare that the research was conducted in the absence of any commercial or financial relationships that could be construed as a potential conflict of interest.
