# Supplementary material for: Genetic Polymorphisms Affecting IDO1 or IDO2 Activity Differently Associate With Aspergillosis in Humans
Source: Front Immunol. 2019 May 7;10:890. doi: 10.3389/fimmu.2019.00890 (PMC6514051; doi:10.3389/fimmu.2019.00890)

SUPPLEMENTARY MATERIALS

Table 1 *IDO1/IDO2* SNPs in both cohorts of CF and BMT patients. The table reports SNP annotation according to Ensembl GRCh37 (hg19) and the Minor Allele Frequency (MAF) for the European population from the 1000 Genomes (MAF_EUR). Hardy-Weinberg p-value (HWE), Genotyping rate (%Geno) and MAF are reported separately for the CF and the BMT patients.

						CF patients			BMT cohort (donors+recipients)		
**#**	**SNP_ID**	**Position hg19**	**Gene**	**Consequence**	**MAF_EUR**	**HWE**	**%Geno**	**MAF**	**HWE**	**%Geno**	**MAF**
1	rs9657182	39765848	*IDO1*	Intron variant	0.456	0.786	99.6	0.420	0.543	72.8	0.445
2	rs3808606	39769375	*IDO1*	Intron variant	0.489	0.908	97.0	0.441	0.657	83.2	0.479
3	rs7820268	39777529	*IDO1*	Intron variant	0.322	0.232	100.0	0.365	0.910	94.2	0.355
4	rs3739319	39785321	*IDO1*	Intron variant	0.391	0.506	98.5	0.419	0.210	74.4	0.422
5	rs16888361	39792914	*IDO2*	Intron variant	0.194	0.790	97.0	0.238	0.594	95.7	0.296
6	rs7846217	39793318	*IDO2*	Intron variant	0.155	0.288	92.6	0.147	0.186	95.1	0.149
7	rs10109853	39862881	*IDO2*	missense variant	0.486	1.000	91.5	0.462	0.042	92.6	0.459
8	rs4503083	39872935	*IDO2*	stop gained	0.211	0.164	92.3	0.256	$6.0x10^{-4}$	94.6	0.196

Raw blots related to Figure 2D

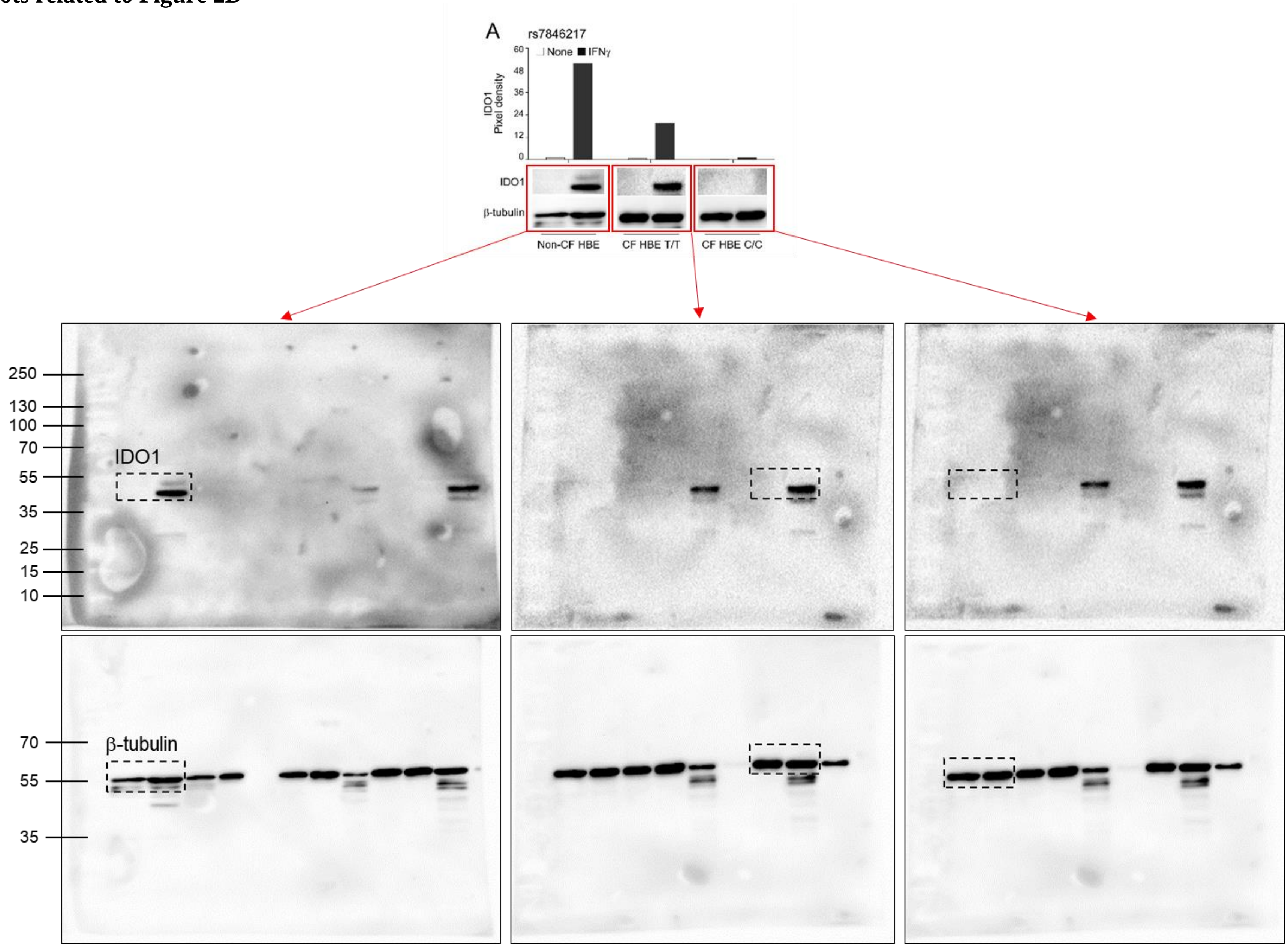

Raw blots related to Figure 3A

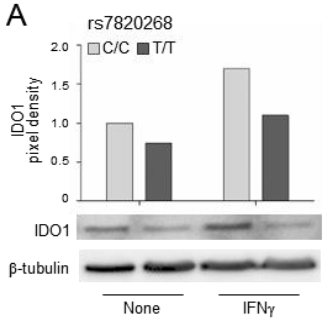

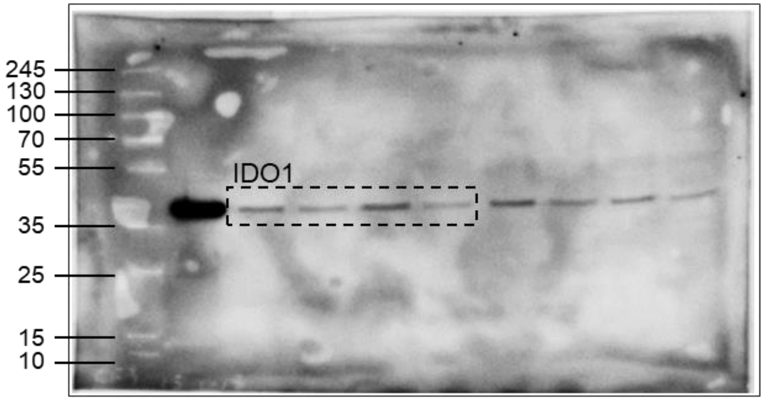

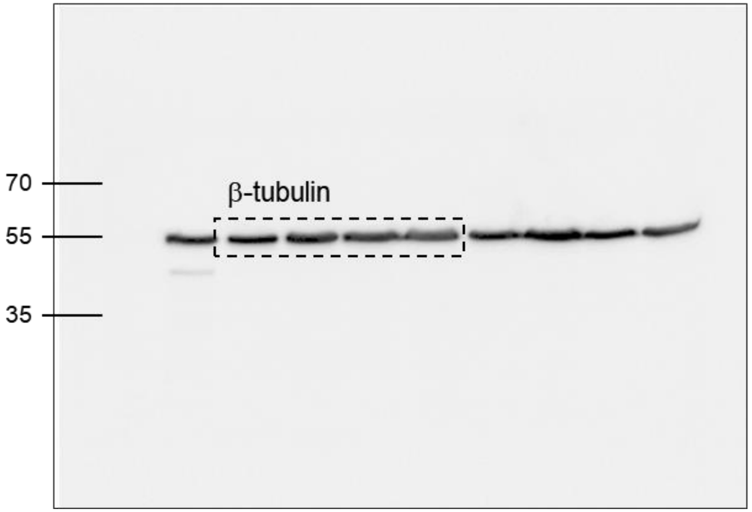

Supplement: Supplementary file 1 [file Data_Sheet_1.PDF]
